# Chronic Pruritus Alleviation in the Elderly Through Drug-Free Autophagy Activation by Magnetized Saline Water: A Case Series

**DOI:** 10.7759/cureus.64428

**Published:** 2024-07-12

**Authors:** Piercarlo Minoretti

**Affiliations:** 1 Occupational Health, Studio Minoretti, Oggiono, ITA

**Keywords:** elderly, skin, autophagy, magnetized saline water, chronic pruritus

## Abstract

Chronic pruritus is a common and distressing condition in the elderly population, frequently associated with various underlying systemic diseases and age-related skin changes. Conventional treatments, such as emollients and moisturizers, may not invariably provide adequate relief. Magnetized saline water has previously been shown to activate autophagy, a cellular process involved in maintaining skin barrier function, reducing inflammaging, and modulating neuropathic pain. This case series investigated the efficacy of a topical serum containing magnetized saline water in managing chronic pruritus with diverse etiologies in elderly patients. Five patients aged 69-80 years, presenting with chronic pruritus lasting two to six months, were instructed to apply the serum daily to the most affected areas for a minimum of 14 consecutive days. Pruritus severity was assessed using the 12-Item Pruritus Severity Scale (12-PSS) at baseline and post-intervention. The underlying causes of pruritus included end-stage renal disease, type 2 diabetes mellitus with peripheral neuropathy, advanced liver fibrosis, and xerosis cutis. All five patients reported a substantial improvement in pruritus severity following the application of the magnetized saline water serum, with post-intervention 12-PSS scores decreasing by 3-5 points. The serum was well-tolerated, and no adverse effects were reported. These findings suggest that topical formulations containing magnetized saline water may be a promising alternative or adjunctive therapy for managing chronic pruritus in the elderly population. However, clinical trials are needed to confirm these findings, elucidate the precise mechanisms of action, and establish optimal treatment protocols.

## Introduction

Chronic pruritus, defined as an unpleasant subjective sensation leading to volitional scratching lasting six or more weeks, can be distressing for patients and challenging for physicians [1]. This condition has a lifetime prevalence of 22-25.5% in the general population [2] and is particularly common in the elderly [3], where it can occur in up to 78% of subjects [4] and be associated with systemic diseases, such as chronic kidney failure, liver diseases, diabetes-associated neuropathy, and hematopoietic disorders [5]. The detrimental effect of pruritus on quality of life is evident and can be associated with anxiety, agitation, difficulty concentrating, and sleep disturbances [2,6].

Xerosis due to an abnormal dermal-epidermal barrier is considered the most common cause of pruritus in geriatric patients [7], although skin inflammaging [8] and neuropathic mechanisms [9] can also play a pivotal role. Autophagy, a highly conserved lysosomal degradation mechanism, plays a pivotal role in maintaining tissue homeostasis by facilitating the recycling of damaged and senescent cellular components [10]. Notably, this process is also implicated in the differentiation of keratinocytes, which are essential for preserving the integrity of the skin barrier function [11]. Impaired autophagy can affect this process, potentially leading to a compromised skin barrier and increased water loss, contributing to xerosis and severe dehydration [12]. Indeed, restoration of the skin barrier using an autophagy activator has been previously shown to increase epidermal tissue integrity ex vivo [13]. In addition, age-related autophagy decline triggers activation of the NLRP3 inflammasome and enhances the inflammaging process [14], a process that impairs skin structure and function in the elderly [8]. Autophagy is also implicated in the pathogenesis of neuropathic pain [15], which shares common mechanisms with neuropathic itch [8], as both conditions are caused by damage or dysfunction in the somatosensory nervous system [8,15]. In this context, Chen et al. [16] previously demonstrated that rapamycin, an autophagy inducer, effectively alleviates allodynia and hyperalgesia in a rat model of neuropathic pain. This finding suggests that modulating autophagy may be a promising therapeutic strategy for managing neuropathic pain and, by extension, neuropathic itch.

Given the potential involvement of impaired autophagy in the development of chronic pruritus in the elderly, we investigated the clinical efficacy of a topical serum containing magnetized saline water, a previously demonstrated activator of skin autophagy [17], in managing chronic itch stemming from various etiologies in geriatric patients.

## Case presentation

Investigational product

The investigational product was a serum (Aquavis, Brescia, Italy) containing 95% magnetized saline water, which has previously been shown to upregulate biomarkers of autophagy in the skin following topical application [17]. Participants who had previously experienced unsuccessful attempts at controlling pruritus with conventional moisturizers or emollients containing ceramides, urea, and/or palmitoylethanolamide were instructed to apply the serum daily to the area most affected by itching for a minimum of 14 consecutive days. All eligible cases were identified consecutively between October 2023 and January 2024. The amount of serum applied was left to the patients’ discretion. They were advised to continue the treatment for at least two weeks or until they observed satisfactory improvement in their pruritus symptoms. If no improvement was noted after the initial 14-day period, participants were instructed to discontinue the use of the product.

Assessment of pruritus severity

The 12-Item Pruritus Severity Scale (12-PSS) is a multidimensional instrument questionnaire designed to comprehensively assess various aspects of pruritus in patients suffering from chronic itch [18]. This questionnaire consists of 12 questions that evaluate the intensity, extent, frequency, duration, and impact of pruritus on daily activities and mood, as well as the patient’s scratching response to the sensation of itch. The final score ranges from 3 to 22 points, with higher scores indicating more severe pruritus. The scale has demonstrated strong psychometric properties, including high internal consistency, satisfactory convergent validity, and a lack of significant ceiling or bottom effects [18]. Based on accepted cut-off values for the 12-PSS, pruritus severity can be categorized as follows: 3-6 points on the 12-PSS (mild pruritus), 7-11 points on the 12-PSS (moderate pruritus), and ≥12 points on the 12-PSS (severe pruritus) [19].

Case report 1

A 75-year-old man with end-stage renal disease who had been undergoing hemodialysis for three years presented with generalized pruritus that persisted for four months. The patient described an unrelenting itching sensation that significantly disrupted his sleep patterns and impaired his overall quality of life. Upon physical examination, the patient exhibited lichenification, particularly prominent on the upper extremities, and excoriations resulting from chronic scratching. To quantify the severity of the patient’s pruritus, the 12-PSS was administered at baseline, yielding a score of 14, which is indicative of severe pruritus. Following a 21-day regimen of nightly topical serum application, the patient reported an alleviation in pruritus intensity and a substantial improvement in sleep quality. The post-intervention 12-PSS score decreased to 9, signifying a transition from severe to moderate pruritus.

Case report 2

A 72-year-old woman with a history of renal failure, managed with peritoneal dialysis for the past 12 months, presented with a chief complaint of pruritus persisting for two months. Her baseline 12-PSS score was 10, indicative of moderate pruritus. The patient subjectively reported bothersome itching, particularly affecting the back and lower extremities, with exacerbation noted upon exposure to heat and during episodes of sweating. Following a three-week treatment course, the patient’s post-intervention 12-PSS score decreased to 6, demonstrating a marked improvement in pruritus severity, now classified as mild.

Case report 3

A 75-year-old female patient with a history of type 2 diabetes mellitus and peripheral neuropathy presented with a chief complaint of pruritus persisting for five months. The patient described a distressing combination of burning and tingling sensations accompanied by intense itching, primarily affecting the lower extremities. Upon initial assessment, the patient’s baseline 12-PSS score was found to be 13, signifying severe pruritus. The topical serum was initiated for a duration of three weeks. Following the treatment course, the patient reported substantial alleviation of pruritus. However, it is noteworthy that the neuropathic sensations, such as burning and tingling, did not demonstrate improvement. The post-intervention 12-PSS score decreased to 9, reflecting an improvement in pruritus severity from severe to moderate.

Case report 4

A 69-year-old woman with a history of advanced liver fibrosis due to chronic hepatitis C and comorbid alcoholic liver disease presented with a four-month history of pruritus. Laboratory findings revealed markedly elevated alanine transaminase (174 U/L; reference range, 0-55 U/L) and aspartate transaminase (126 U/L; reference range, 0-34 U/L), cholestasis indices (alkaline phosphatase, 322 U/L; reference range, 40-150 U/L; gamma-glutamyl transferase, 168 U/L; reference range, 12-64 U/L), mild hyperbilirubinemia (bilirubin, 2.2 mg/dL; reference range, 0.3-1.2 mg/dL) without clinical jaundice, and hypoalbuminemia (albumin, 3.7 g/dL; reference range, 6.4-8.3 g/dL). The patient reported generalized itching, worsening at night, and significantly impacting sleep quality. The baseline 12-PSS score was 12, indicating severe pruritus. After five weeks of serum application, the patient reported improved pruritus intensity and sleep quality. The post-intervention 12-PSS score decreased to 9, indicating improvement but remaining in the moderate pruritus category.

Case report 5

An 80-year-old man presented with a six-month history of pruritus associated with xerosis cutis. The patient reported persistent, diffuse pruritus that was exacerbated by low ambient humidity. Physical examination revealed xerotic, scaling skin with visible excoriations secondary to chronic scratching. The patient’s baseline 12-PSS score was 9, indicating moderate pruritus. After four weeks of nightly application of the study serum, the patient reported subjective improvement in skin hydration and reduced pruritus intensity. The post-intervention 12-PSS score decreased to 6, reflecting an improvement from moderate to mild pruritus severity. A summary of the five reported cases is provided in Table 1.

**Table 1:** General characteristics of the five patients described in the case series

Patient number	Age (years) and sex	Cause of pruritus	Duration of pruritus, months	12-Item Pruritus Severity Scale, baseline score	Duration of serum application, weeks	12-Item Pruritus Severity Scale, post-intervention score
1	75, male	End-stage renal disease, hemodialysis	4	14	3	9
2	72, female	Renal failure, peritoneal dialysis	2	10	3	6
3	75, female	Type 2 diabetes mellitus, peripheral neuropathy	5	13	3	9
4	69, female	Advanced liver fibrosis, chronic hepatitis C, alcoholic liver disease	4	12	5	9
5	80, male	Xerosis cutis	6	9	4	6

## Discussion

Emollients are commonly prescribed as the first-line topical treatment for chronic pruritus in individuals over 65 years of age [4]. Alternative treatments, such as topical corticosteroids and calcineurin inhibitors, are used less frequently [4]. When an underlying causative disease for itching is identified, it should also be treated appropriately [5]. The primary pathophysiological mechanisms underlying pruritus in the elderly include dermal-epidermal barrier dysfunction [7], skin inflammaging [8], and neuropathic processes [9]. Given that autophagy plays a crucial role in all three of these mechanisms [13-15], this case series evaluated the efficacy of a serum formulation containing magnetized saline water in improving pruritus. Recently, magnetized saline water has emerged as a promising non-pharmacological approach to activate autophagy [17,20]. In this study, we present five elderly patients, each with pruritus of various etiologies that had been refractory to conventional moisturizers and emollients. The application of a magnetized water serum yielded promising results in all cases, as evidenced by a consistent reduction in 12-PSS scores. In addition, the serum was well-tolerated, and no adverse effects were reported. These findings demonstrate the potential effectiveness of magnetized water-based topical formulations in the management of chronic pruritus in the elderly population.

The precise mechanisms by which magnetized saline water may alleviate pruritus were not specifically investigated in this study. However, we hypothesize that they may involve activation of the autophagy flux, with consequent improvement in skin biophysical parameters [17]. Previous research has shown that magnetized saline water applied topically in a vehicle can lead to an increased expression of autophagy-related biomarkers [17,20]. By activating autophagy in the skin, the magnetized water serum may help restore barrier integrity and reduce inflammaging, thereby alleviating pruritus. It is also plausible that the modulation of autophagy can alleviate neuropathic pain by promoting neuronal homeostasis and survival in the somatosensory nervous system [15,16]. In addition to activating autophagy, magnetized water exhibits several unique properties compared to regular water, including increased electrical conductivity, lower surface tension, and reduced dissolved oxygen levels [21]. These specific properties may contribute to the beneficial effects observed in this case series, potentially by enhancing skin hydration or modulating the skin microenvironment.

While the results of this case series are encouraging, it is essential to recognize the inherent limitations of the study design. The small sample size and the lack of a control group are significant caveats that should be considered when interpreting the findings. Furthermore, although the 12-PSS provides a comprehensive assessment of pruritus, including intensity, extent, duration, impact on quality of life, and scratching response [18,19], it can be argued that the 5-D itch scale [22] may be more specific for evaluating itch distribution. The 5-D itch scale is also more concise, comprising only five domains [22], and can be completed more quickly. However, it is important to note that both scales have demonstrated high reliability and validity for assessing chronic pruritus [18,19,22]. An intriguing observation from this case series is that the longest period of serum use (five weeks) was observed in a patient with liver pathology, while shorter treatment durations were sufficient to achieve positive effects in cases of other pathologies. The pathophysiological basis for this observation remains unclear but may suggest that pruritus associated with chronic hepatic disorders may be more challenging to treat using topical autophagy activators. Further research is needed to elucidate the underlying mechanisms and optimize treatment strategies for pruritus related to specific pathologies.

It is also noteworthy that all patients in this case series requested to continue the topical treatment, indicating a high level of patient satisfaction with the serum’s effectiveness in managing their symptoms. The positive response to the serum and the desire for ongoing use suggest that the participants found the treatment to be an effective long-term management strategy for their pruritus. However, definitive conclusions about the durability of the effects upon treatment discontinuation cannot be drawn from this study, as this would require a controlled discontinuation of the treatment and long-term follow-up assessments. To further evaluate the efficacy and safety of magnetized water-containing topical formulations for the treatment of pruritus in elderly populations, larger, well-designed, randomized controlled trials are necessary.

## Conclusions

This case series provides preliminary evidence supporting the use of a magnetized saline water serum as a novel treatment option for chronic pruritus in elderly patients. Given the challenges associated with managing pruritus in this population and the potential for adverse effects with conventional therapies, magnetized water-based topical formulations may represent a promising alternative or adjunctive approach. However, further research is warranted to confirm these findings, establish optimal treatment protocols, and ensure maximum efficacy and safety. As the scientific community continues to explore innovative solutions for managing chronic pruritus in the elderly, it is essential to prioritize patient well-being and quality of life while rigorously evaluating the benefits and risks of emerging therapies.
